# Deficit of Mitonuclear Genes on the Human X Chromosome Predates Sex Chromosome Formation

**DOI:** 10.1093/gbe/evv017

**Published:** 2015-01-29

**Authors:** Rebecca Dean, Fabian Zimmer, Judith E. Mank

**Affiliations:** Department of Genetics, Evolution and Environment, University College London, United Kingdom

**Keywords:** sexual conflict, sex chromosomes, mitochondria, synteny

## Abstract

Two taxa studied to date, the therian mammals and *Caenorhabditis elegans*, display underrepresentations of mitonuclear genes (mt-N genes, nuclear genes whose products are imported to and act within the mitochondria) on their X chromosomes. This pattern has been interpreted as the result of sexual conflict driving mt-N genes off of the X chromosome. However, studies in several other species have failed to detect a convergent biased distribution of sex-linked mt-N genes, leading to questions over the generality of the role of sexual conflict in shaping the distribution of mt-N genes. Here we tested whether mt-N genes moved off of the therian X chromosome following sex chromosome formation, consistent with the role of sexual conflict, or whether the paucity of mt-N genes on the therian X is a chance result of an underrepresentation on the ancestral regions that formed the X chromosome. We used a synteny-based approach to identify the ancestral regions in the platypus and chicken genomes that later formed the therian X chromosome. We then quantified the movement of mt-N genes on and off of the X chromosome and the distribution of mt-N genes on the human X and ancestral X regions. We failed to find an excess of mt-N gene movement off of the X. The bias of mt-N genes on ancestral therian X chromosomes was also not significantly different from the biases on the human X. Together our results suggest that, rather than conflict driving mt-N genes off of the mammalian X, random biases on chromosomes that formed the X chromosome could explain the paucity of mt-N genes in the therian lineage.

## Introduction

A series of studies have recently generated substantial debate over the role of intergenomic conflict in driving mitonuclear (mt-N) gene distributions on and off sex chromosomes (Drown et al. 2012; Hill and Johnson 2013; Dean et al. 2014; Hough et al. 2014; Rogell et al. 2014). mt-N genes are loci whose products, encoded by the nuclear genome, are then imported into the mitochondria, which is the primary site of their activity. Because mitochondria and sex chromosomes have different inheritance patterns between the sexes, intergenomic conflict has been suggested as a potential explanation for the underrepresentation of mt-N genes on the X chromosomes of some animals (Drown et al. 2012; Dean et al. 2014). Mitochondria are maternally inherited in many species (although low rates of male transmission may occur, e.g., Wolff et al. 2013), and are therefore selected for female fitness effects, as male mitochondria are generally evolutionary dead ends. It has been shown that maternal transmission of mitochondria can result in quite serious costs to males, through the disruption of male function (Partridge and Hurst 1998; Innocenti et al. 2011; Drown et al. 2012).

The accumulation of mutations that are detrimental to males could be ameliorated if genes that interact with the mitochondria move to a more favorable genomic location for the evolution of compensatory mechanisms. Genes on the X chromosome, which spend two-thirds of their time in females, are more often cotransmitted with mitochondria than autosomal genes (Rand et al. 2001), and the X chromosome is also feminized in several species (reviewed in Dean and Mank 2014). This might make the X chromosome particularly unfavorable for male-biased compensation of the mitochondrial mutational load. It is therefore possible that there has been selection in males for the movement of mt-N genes off of the X chromosome in order to reduce disruption to male function induced by maternally transmitted mitochondria.

Consistent with the conflict hypothesis, *Caenorhabditis elegans* (Dean et al. 2014) and the therian mammals (Drown et al. 2012) show a deficit of mt-N genes on their X chromosomes, and genes sensitive to mitochondrial polymorphism are scarce on the *Drosophila* X chromosome (Rogell et al. 2014). However, a broader phylogenetic assessment of mt-N gene distributions revealed a mixed pattern, with most male heterogametic species studied showing no significant bias (Dean et al. 2014; Hough et al. 2014). Moreover, many sex-specific evolutionary properties observed on the X chromosome are observed in converse on Z chromosomes, such as distributions of sex-biased genes (Arunkumar et al. 2009; Wright et al. 2012), so we might expect a corresponding overabundance of Z-linked mt-N genes in female heterogametic systems; however, no such overabundance has yet been observed (Dean et al. 2014). Furthermore, if conflict is at least partly responsible for the genomic distribution of mt-N genes, it might also be expected to shape the distribution of nuclear genes that interact with the chloroplast, which is also often maternally inherited, but no bias was detected in the distribution of chloro-nuclear genes on the X chromosome in *Rumex* (Hough et al. 2014), a dioecious plant with sex chromosomes.

These patterns of mt-N gene distributions suggest that either conflict is particularly strong only in therian mammals and nematodes, or that some effect other than conflict explains the distribution in these two clades. The incorporation of mitochondrial loci into the nuclear genome began long before the formation of sex chromosomes in any single extant lineage (Dyall et al. 2004; Timmis et al. 2004; Cortez et al. 2014) and strong chromosomal biases exist for many autosomes, presumably due to chance variation in gene content (Drown et al. 2012; Dean et al. 2014; Hough et al. 2014). This presents the possibility that biases in mt-N gene distributions need not be driven by conflict, but instead could predate the formation of the sex chromosome, if the precursor autosomes showed an ancestral bias through chance alone.

We tested whether ancestral gene distributions can explain the underrepresentation of mt-N genes on therian sex chromosomes. The rapid gene and genome evolution in *Caenorhabditis* (Lipinski et al. 2011) precludes reconstruction of syntenic relationships across even closely related species, but amniotes have strongly conserved synteny (Dehal and Boore 2005), making it possible to identify syntenic regions in divergent taxa. In order to determine whether the paucity of mt-N genes on the therian X chromosome is a consequence of intergenomic sexual conflict, or whether it is simply the product of a biased distribution on the ancestral autosome that gave rise to the therian X chromosome, we tested the mt-N gene distributions on the ancestral regions syntenic to the therian X in platypus and chicken (hereafter termed X-syntenic regions).

We used the human X chromosome as our point of reference because of its excellent annotation. As the human X is broadly syntenic across therian mammals (Ohno 1967; Murphy et al. 1999; Band et al. 2000; Raudsepp et al. 2004), it is representative of the therian X in general. We identified regions in synteny with the human X in platypus (*Ornithorhynchus anatinus*) and chicken (*Gallus gallus*), the most recent ancestors to the Theria with different sex chromosomal systems (Graves 2006) and annotated genomes. This enabled us to use two complementary approaches to test the role of conflict in driving mt-N gene distributions. First, we identified orthologous genes, in platypus and chicken, to the human mt-N genes. We then tested for an excess of mt-N gene movement in order to investigate whether intergenomic conflict has driven mt-N genes off of the human X following sex chromosome formation. Second, we used these orthologous genes to compare mt-N gene distributions on human X and X-syntenic regions in platypus and chicken. If the abundance of mt-N genes on the X-syntenic regions is more than the abundance on the human X, then intergenomic conflict may have driven mt-N genes off of the therian X following sex chromosome formation. If, on the other hand, mt-N biases on the ancestral autosomes that gave rise to the therian X chromosome show a similar underrepresentation to the human X, then the chromosomal bias is unlikely to be a consequence of intergenomic conflict and may simply be a result of random variation across chromosomes in mt-N content.

## Results and Discussion

### mt-N Gene Movement On and Off the Human X Chromosome

**Fig. 1.—:**
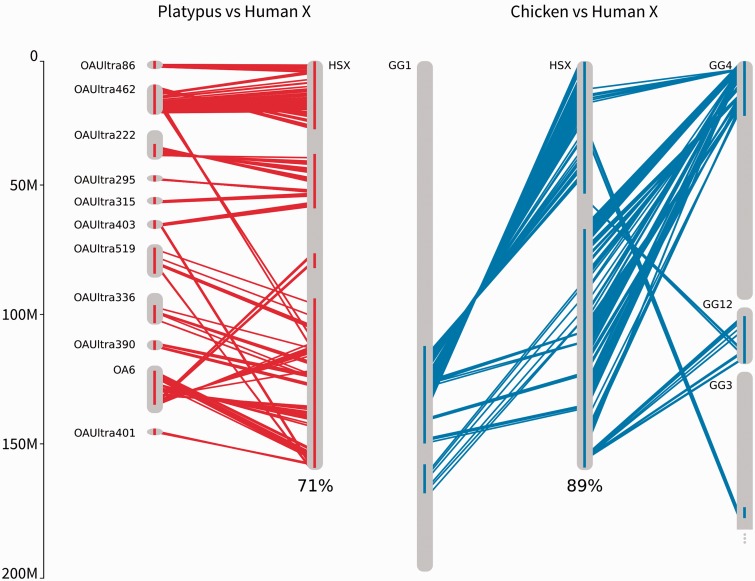
Syntenic regions between (*a*) human X (HSX) and platypus chromosome 6 (OA6) and several unmapped contigs (OAUltra) and (*b*) human X (HSX) and chicken chromosomes 1 (GG1), 4 (GG4), 3 (GG3), and 12 (GG12). Lines represent genes in synteny, red for platypus to human, blue for chicken to human. Blocks on chromosomes show regions where single MCScanX alignments are located on the chromosome closer than 10 million base pairs.

We identified platypus chromosome 6 plus ten unmapped ultracontigs (platypus hX-syntenic regions), and regions of chicken chromosomes 1, 3, 4 and 12 (chicken hX-syntenic regions), as syntenic with the human X chromosome (fig. 1). The platypus hX-syntenic regions comprised a total of 381 genes spanning 71% of the length of the human X-chromosome and the chicken hX-syntenic regions comprised a total of 908 genes spanning 89% of the length of the human X-chromosome (fig. 1). The reduced coverage of the human X chromosome in platypus is largely due to the poorer assembly of the platypus genome.

To test whether an excess of mt-N gene movement off of the human X chromosome occurred following human X chromosome formation, we identified the location of the human mt-N orthologs in platypus and chicken. Pairs of orthologous genes that did not fall within syntenic blocks were potential candidates for genes that have moved. We identified four genes that moved onto the human X from Ultra contigs that were not in platypus hX-syntenic regions (from UltraContig 369; UltraContig 98; and two genes from UltraContig 519) and no genes that might have moved off the human X. These numbers were not significantly different than what we would expect based on the relative size and content of the X chromosome (Betrán et al. 2002; Vibranovski et al. 2009; Toups et al. 2011; Fisher’s exact test, *P* > 0.6), suggesting no excess of gene movement onto or off of the human X chromosome (table 1*a*). However, two of the genes that might have moved onto the X were from UltraContig 519, part of which constitutes the platypus hX-syntenic region. Removing these genes does not qualitatively affect our results (Fisher’s exact test, *P* > 0.2).

**Table 1:** Movement of mt-N Genes On and Off the X between (*a*) Platypus and Human and (*b*) Chicken and Human

Movement	Observed	Expected^a^
(*a*) Platypus → human		
X → A	0	2
A → X	4	4
A → A	132	130
	*P* = 0.640	
(*b*) Chicken → human		
X → A	3	4
A → X	3	4
A → A	92	90
	*P* = 0.845	

Note.—X → A is hX-syntenic to autosome; A → X is autosome to human X syntenic region; A → A is autosome to autosome. *P* value is from Fisher’s exact test.

^a^Calculated based on relative size and content of the X chromosome (Betrán et al. 2002; Vibranovski et al. 2009; Toups et al. 2011).

Between human and chicken, we identified three genes that moved onto the X (from GG8 and two from GG4) and three genes that moved off the X (to HS3 and two to HS2). This is not greater than what we would expect based on the size of the X chromosome (Fisher’s exact test, *P* > 0.8, table 1*b*). Again, two of the genes that may have moved onto the X came from regions of GG4 that were close to the hX-syntenic region. These gene movements do not suggest an excess of mt-N gene movement off the human X (table 1*b*, excluding two genes that might not have moved onto the X, Fisher’s exact test, *P* > 0.3). One of these genes (ENSP00000362773) was also found to move onto the X in platypus (platypus UltraContig 369 to HSX; chicken GG4 to HSX).

### mt-N Gene Abundance on X Syntenic Regions

**Fig. 2.—:**
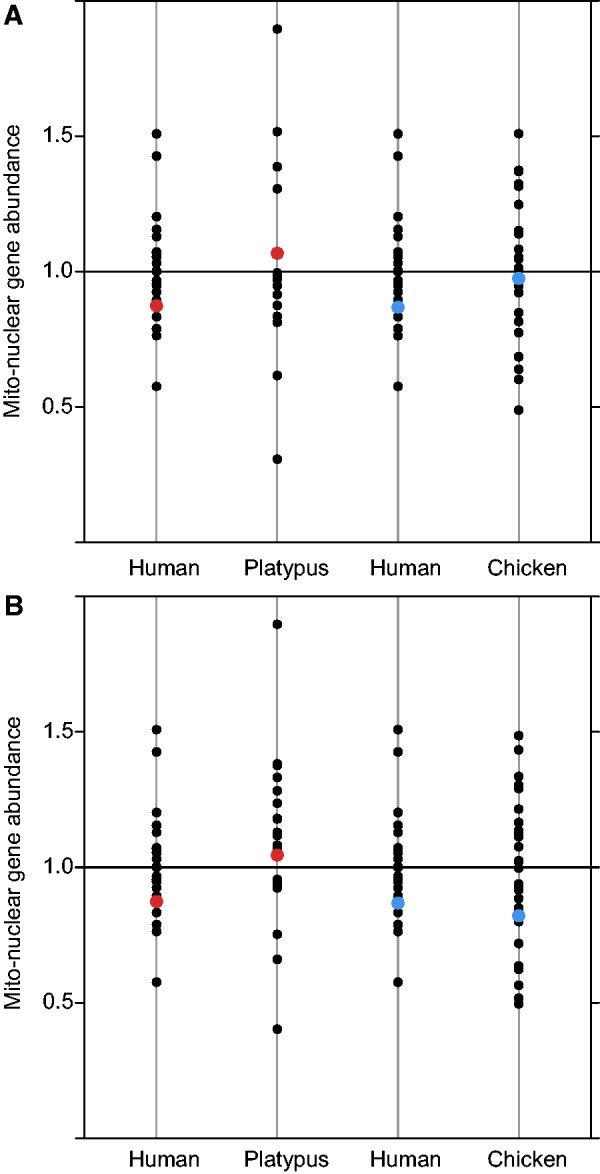
Bias of mt-N genes in human, platypus, and chicken. Autosomes in black and hX-syntenic regions with platypus in red, hX-syntenic regions with chicken in blue. (*a*) mt-N genes are inferred using orthology with human mt-N genes, and total gene counts include only those genes that are orthologous between human and platypus or human and chicken. (*b*) mt-N genes are inferred through species-specific annotations in Biomart and gene counts are all annotated genes.

Our second approach was to compare the abundance of mt-N genes on human X chromosome regions that were syntenic to the identified regions in platypus and chicken. The bias (a measure of mt-N gene density, see Materials and Methods) of mt-N genes does not differ between human X and platypus hX-syntenic regions (Fisher’s exact two-tailed test, *P* = 0.616; fig. 2*a*, table 2) or human X and chicken hX-syntenic regions (Fisher’s exact two-tailed test, *P* = 0.793; fig. 2*a*, table 2), suggesting that the cause of the underrepresentation on the human X is more likely the result of a random underrepresentation of mt-N genes on the chromosomal regions that formed the human X, rather than intergenomic conflict driving genes off of the X after its formation. We also calculated mt-N gene abundances using species-specific Gene Ontology annotation (GO:0005739) in Biomart to identify mt-N genes. The two approaches to infer mt-N gene function largely agree (platypus 76% overlap; chicken 82% overlap), hence calculating mt-N abundance using Biomart gave qualitatively similar results (table 2, fig. 2*b*, human X and platypus hX-syntenic region, Fisher’s exact test, *P* = 0.719; human X and chicken hX-syntenic regions, Fisher’s exact test, *P* = 0.893).

**Table 2:** Number of mt-N, Total Number of Genes, and the Bias in Distribution of mt-N Genes on the Human X and X-Syntenic Regions Using Gene Orthology to Identify mt-N Genes and Using Species-Specific mt-N Gene Annotations in Biomart

Species	mt-N Genes	Total Genes	Bias	95% CI
Human X	55	820	0.85	0.64–1.06
Platypus hX-syntenic (orthology)	29	309	1.07	0.70–1.43
Platypus hX-syntenic (biomart)	23	381	1.05	0.63–1.45
Human X (syntenic platypus)	46	667	0.87	0.63–1.12
Chicken hX-syntenic (orthology)	64	727	0.97	0.75–1.20
Chicken hX-syntenic (biomart)	52	908	0.83	0.60–1.04
Human X (syntenic chicken)	49	715	0.87	0.64–1.10

Note.—Gene counts are for the hX-syntenic blocks, the boundaries of which are created by merging alignments when alignments were closer than 10 million base pairs. The greater number of orthologous genes on chicken hX-syntenic than on the human X syntenic with chicken region is a consequence of these merged alignments.

### Gene Annotation and mt-N Abundance

The measure of abundance (bias) relies on the total number of mt-N genes and total number of genes annotated in each species. This means that measures of bias are susceptible to variation in the quality of genome annotation. The underrepresentation of mt-N genes on the whole of the human X in this study is 0.86 ± 0.22 (bias ± 95% CI), which is less pronounced than the underrepresentation previously reported for the human X chromosome (Drown et al. 2012; Dean et al. 2014). The human genome assembly version has recently been updated from GrCH37 to GrCH38, resulting in changes to the total number of genes and number of mt-N genes, which can account for the different mt-N bias on the human X (bias ± 95% CI, 0.76 ± 0.21 using GrCH37). Gene annotation quality also likely accounts for the overabundance of mt-N genes on the platypus hX-syntenic regions (29 observed mt-N genes and 25 expected), despite a lack of mt-N gene movement off of the X chromosome following X chromosome formation.

### mt-N Gene Abundance across Independent X Chromosomes

Across the seven independent X chromosomes studied to date, two (therian mammals and *C. elegans*) show a significant underrepresentation of mt-N genes, three (*Rumex*, platypus and stickleback) exhibit a nonsignificant underrepresentation, and two (*Tribolium* and *Drosophila*) show a nonsignificant overrepresentation (Drown et al. 2012; Dean et al. 2014; Hough et al. 2014). This does not represent a significant overall underrepresentation of mt-N genes on X chromosomes (two-tailed sign-test; 5 of 7, *P* = 0.453). If the distribution of mt-N genes on X chromosomes is explained by variation in ancestral autosomes, we would expect both under- and overrepresentations of mt-N genes on X chromosomes. This is consistent with what we find; however, our ability to detect a significant widespread underrepresentation (i.e., the signature of conflict) is not particularly powerful, with only seven different X chromosomes having been quantified so far. An alternative explanation is that mt-N interactions predispose chromosomes depauperate of mt-N genes to become sex chromosomes, although this predisposition might be rather weak and highly dependent upon the location of genes involved in sex determination.

## Conclusion

Our results suggest that the underrepresentation of mt-N genes on the therian X is not a result of gene movement off of the X chromosome. Rather, the paucity of mt-N genes on the therian X predates the formation of the therian sex chromosomes, and selection has acted mainly to maintain this ancestral distribution after sex chromosome formation. Even though we find no support for conflict driving mt-N genes off the therian X chromosome, random genomic biases in mt-N gene distributions could have important consequences for mt-N coadaptation and potentially for sex chromosome formation. A paucity of mt-N genes on the therian X chromosome means that genes that interact with the mitochondria are less often cotransmitted compared with mt-N genes on autosomes. This might affect rates of coevolution between mitochondria and nuclear genes (e.g., Hill 2014), with possible fitness consequences (Montooth et al. 2010; Meiklejohn et al. 2013).

## Materials and Methods

### Identification of Ancestral Chromosomes to the Human X Chromosome through Whole-Genome Synteny Analysis

In the first step, we obtained the human (*Homo sapiens*), platypus (*Ornithorhynchus anatinus*), and chicken (*Gallus gallus*) proteomes from Ensembl version 76 (Flicek et al. 2014). We used the longest isoforms as input for BLASTP (Altschul et al. 1990) to detect homologs between the human proteome and both platypus (supplementary table S1, Supplementary Material online) and chicken (supplementary table S2, Supplementary Material online) (*e* value < 10^−^^10^). We then used the BLASTP output and positional information as input for MCScanX (Wang et al. 2012), used with default values, to detect homologous chromosomal regions between human and platypus (supplementary table S3, Supplementary Material online) and human and chicken (supplementary table S4, Supplementary Material online). Only genes that have been mapped to a chromosome were included for human and chicken; genes on UltraContigs were included for platypus, as a larger proportion of this genome assembly is currently mapped to scaffolds and contigs rather than chromosomes. The homologous chromosomal regions of the human X chromosome on platypus and chicken chromosomes were identified as the ancestral chromosomes to the human X chromosome. If the individual MCScanX alignments were closer than 10 million base pairs, we merged the alignments into a larger syntenic region to reflect the process of chromosome rearrangement (Burt et al. 1999; Coghlan et al. 2005) and sex chromosome formation (Lahn and Page 1999).

### Identification of mt-N Gene Movement

Mt-N genes were identified in human using Gene Ontology annotation (GO:0005739) in Biomart Ensembl Genes 76. To track movement of mt-N genes on and off the X we identified one-to-one orthologs of the 1,572 human mt-N genes in platypus and chicken using reciprocal best hit BLAST (rBBH), with a minimum *e* value of 10^−^^10^. Significant hits were ordered by bitscore and a rBBH was only counted when the tophit had a sequence identity larger than 30%. This resulted in 1,064 rBBH between human and platypus, and 1,116 between human and chicken. Of those, 575 rBBH between human and platypus, and 1,087 between human and chicken, were on a sufficiently large scaffold to infer synteny (i.e., Ultra contigs in platypus and chromosomes in chicken).

To identify whether movement of mt-N genes on and off of the X chromosome represents an excess of gene movement, we calculated the expected number of movements based upon the number of genes on source chromosomes and the number of base pairs on the target chromosomes (Betrán et al. 2002; Vibranovski et al. 2009; Toups et al. 2011). Fisher’s exact two-tailed tests were used to test whether observed movements were different from expected.

### mt-N Abundance

Gene counts of protein-coding genes were calculated using Biomart Ensembl Genes 76. When comparing the abundance of mt-N genes on ancestral X and therian X between species, we used only the regions of the human X chromosome that were identified as syntenic in the other species. The bias of the distribution of mt-N genes on the human X and the platypus and chicken X-syntenic regions was calculated as: Bias =number of mt-N genes/expected number of mt-N genes, where the expected number was calculated as: Expected number = (number of genes in region/total genes) × total mt-N genes.

Mt-N genes in platypus and chicken were identified using two approaches, first, using the orthologous genes to the mt-N genes in human and second, using species-specific Gene Ontology annotation (GO:0005739) in Biomart Ensemble Genes 76. In chicken and platypus GO:0005739 genes are inferred from electronic annotation (evidence code IEA), which includes sequence similarity, database records, and keyword mapping files. As such, the orthology approach and the Biomart approach to infer gene function largely agree, with 76% overlap between the two approaches for platypus and 82% overlap for chicken.

Confidence intervals were calculated using 10,000 bootstrapped samples by randomly sampling genes with replacement and calculating the bias for each iteration. Differences between the expected and actual number of mt-N genes on the human X and platypus or chicken X-syntenic regions were calculated using a Fisher’s exact test. Analyses were conducted in R v2.15.1 (R Development Core Team, 2013) 

## Supplementary Material

Supplementary tables S1–S4 are available at *Genome Biology and Evolution* online (http://www.gbe.oxfordjournals.org/).

Supplementary Data
