# Supplementary material for: Deficit of Mitonuclear Genes on the Human X Chromosome Predates Sex Chromosome Formation
Source: Genome Biol Evol. 2015 Jan 29;7(2):636–41. doi: 10.1093/gbe/evv017 (PMC4350183; doi:10.1093/gbe/evv017)
Supplement: Supplementary Data [file supp_7_2_636__index.html]

Deficit of Mitonuclear Genes on the Human X Chromosome Predates Sex Chromosome Formation — Supplementary Data 

# Deficit of Mitonuclear Genes on the Human X Chromosome Predates Sex Chromosome Formation

## Supplementary Data

files

**Files in this Data Supplement:**

- Supplementary Data - tsv file
- Supplementary Data - tsv file
- Supplementary Data - tsv file
- Supplementary Data - tsv file
